# Supplementary material for: Side chain to main chain hydrogen bonds stabilize a polyglutamine helix in a transcription factor
Source: Nat Commun. 2019 May 2;10:2034. doi: 10.1038/s41467-019-09923-2 (PMC6497633; doi:10.1038/s41467-019-09923-2)
Supplement: Supplementary file 1 — Supplementary Information [file 41467_2019_9923_MOESM1_ESM.pdf]

Side chain to main chain hydrogen bonds stabilize a polyglutamine helix in a transcription factor

A. Escobedo, B. Topal et al.

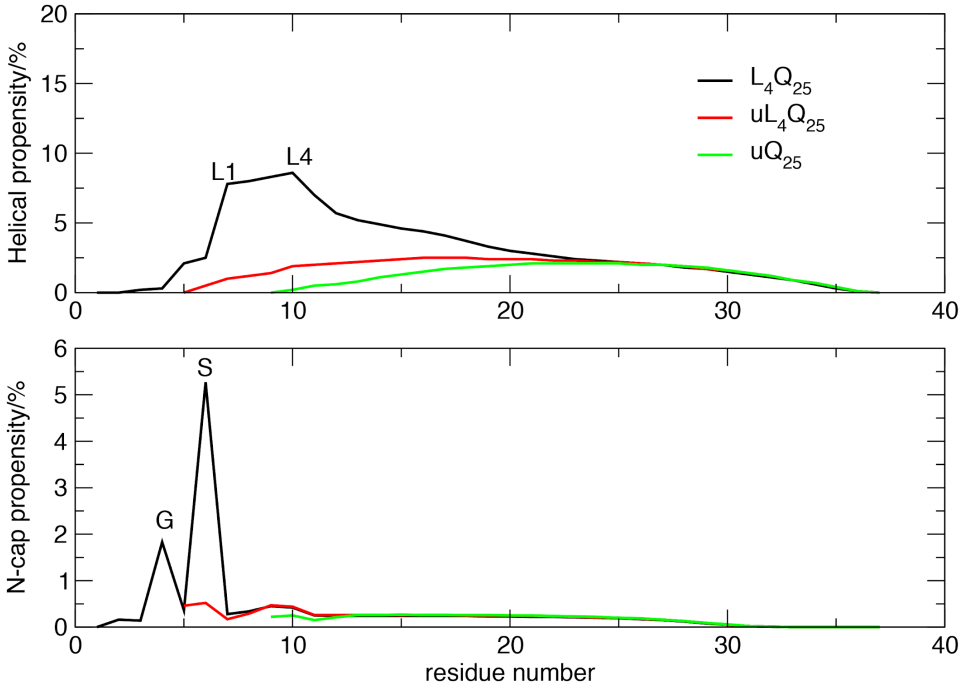

Supplementary Figure 1 | Prediction of helical and N-cap propensity, for peptides uQ_{25}, uL_4Q_{25} and L_4Q_{25} obtained by using Agadir[1]. The peptides have been aligned so that the Gln residues, that are common to all of them, have the same residue numbers (Q11-Q35).

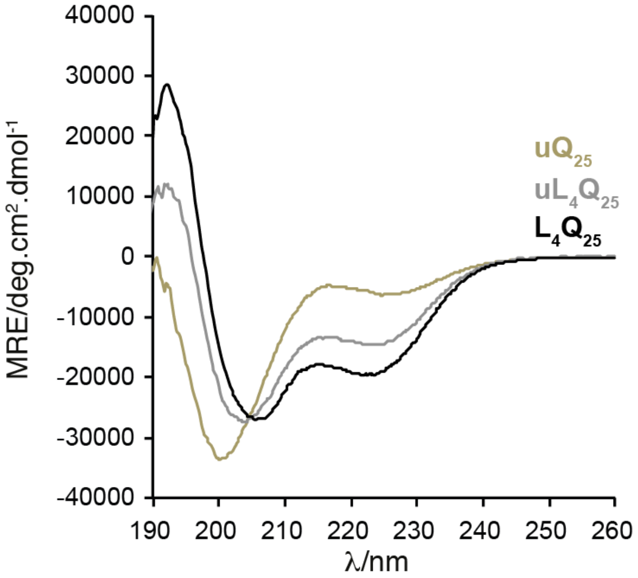

Supplementary Figure 2 | CD spectra of uQ_{25}, uL_4Q_{25} and L_4Q_{25}.

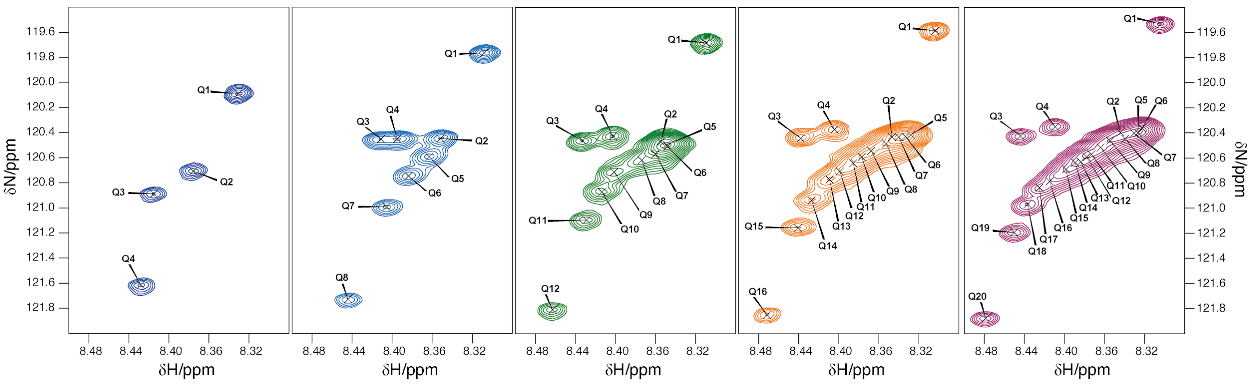

Supplementary Figure 3 | Region of the ^{1}H,^{15}N HSQC spectrum of peptides L_4Q_4 to L_4Q_{20} showing the resonances belonging to the backbone amide groups of Gln residues.

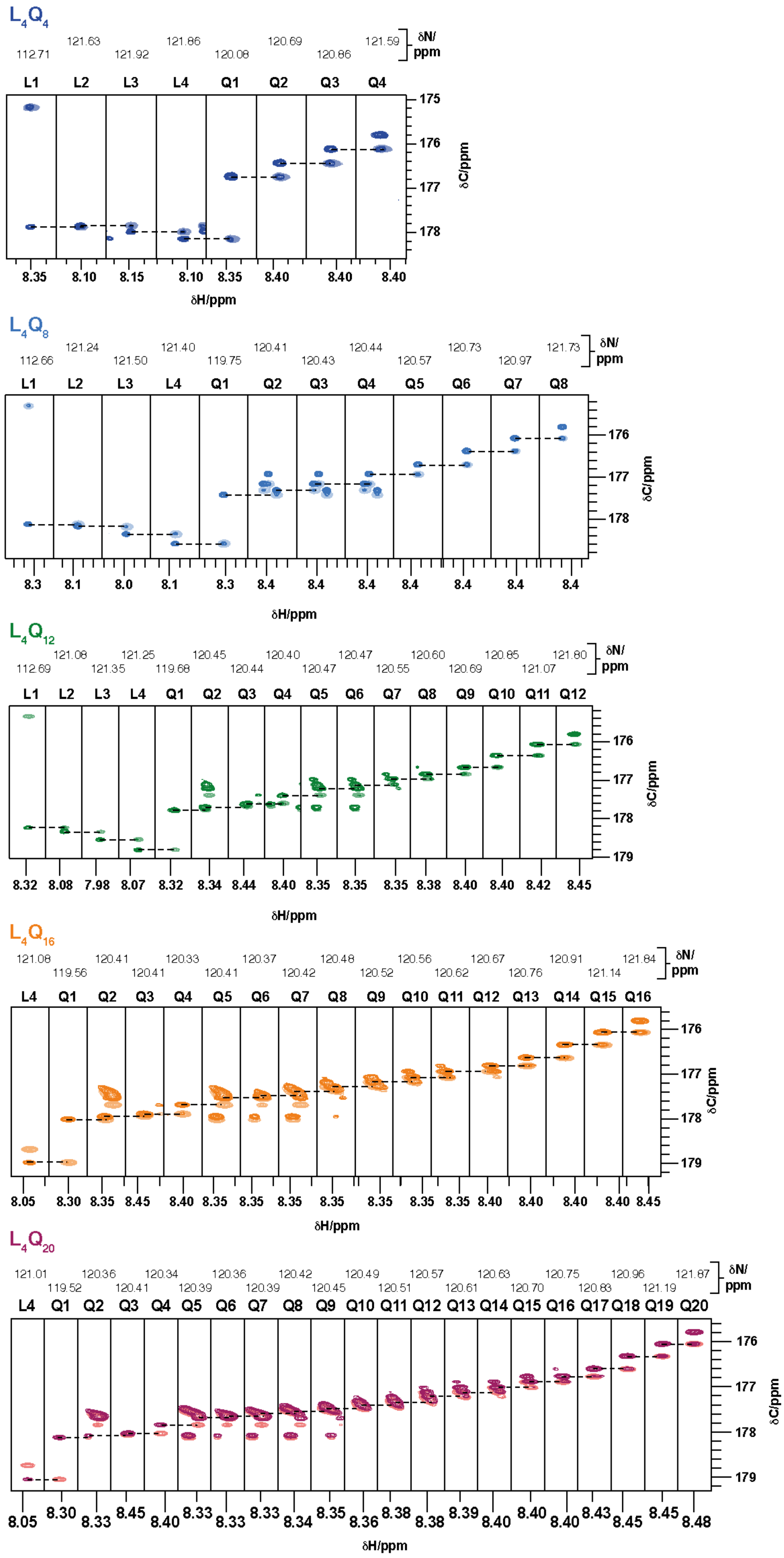

Supplementary Figure 4 | HNCO (light) and HN(CA)CO (dark) spectra of peptides L_4Q_4 to L_4Q_{16} used for the assignment of the backbone resonances.

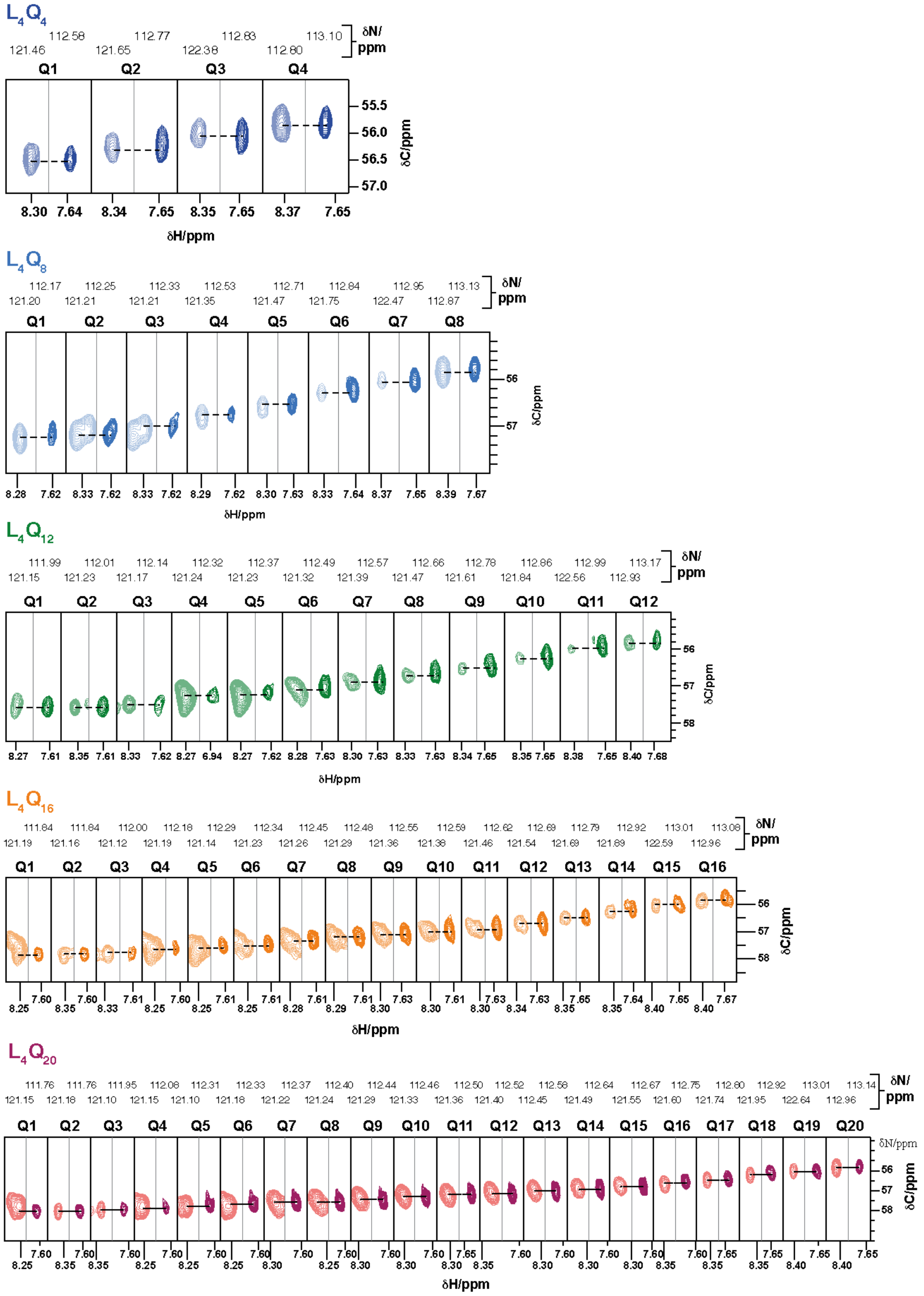

Supplementary Figure 5 | Strip plots showing the Cα cross-peaks correlated to the main chain NH resonances in the HN(CO)CACB experiment (light) and side chain $NH\varepsilon_{21}$ resonances in the (H)CC(CO)NH experiment (dark), used in the assignment of the latter.

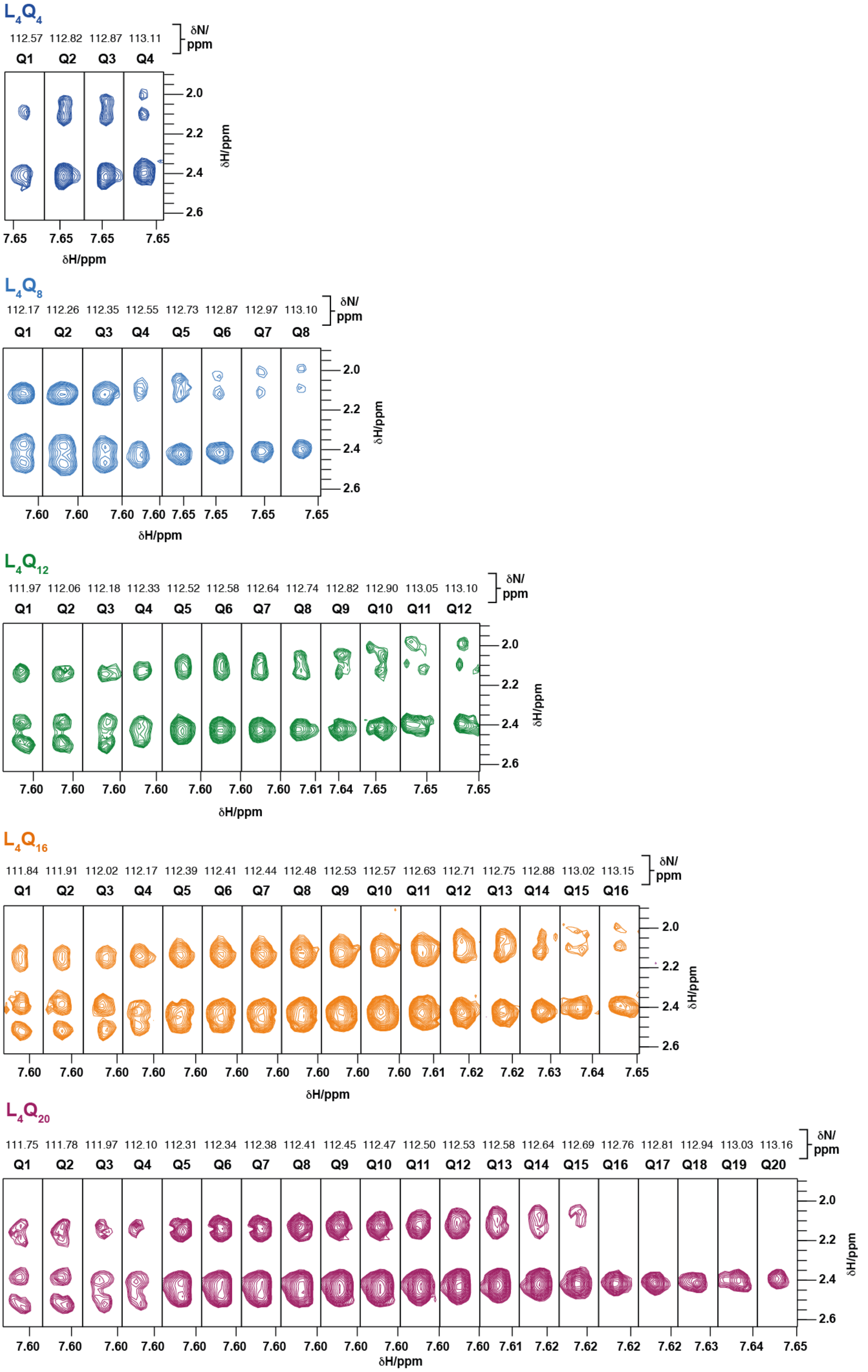

Supplementary Figure 6 | Hε_{21}-centered regions of the ^{15}N planes of the H(CC)(CO)NH spectra of peptides L_4Q_4 to L_4Q_{20} containing the side chain aliphatic ^{1}H resonances of all residues of the polyQ tract.

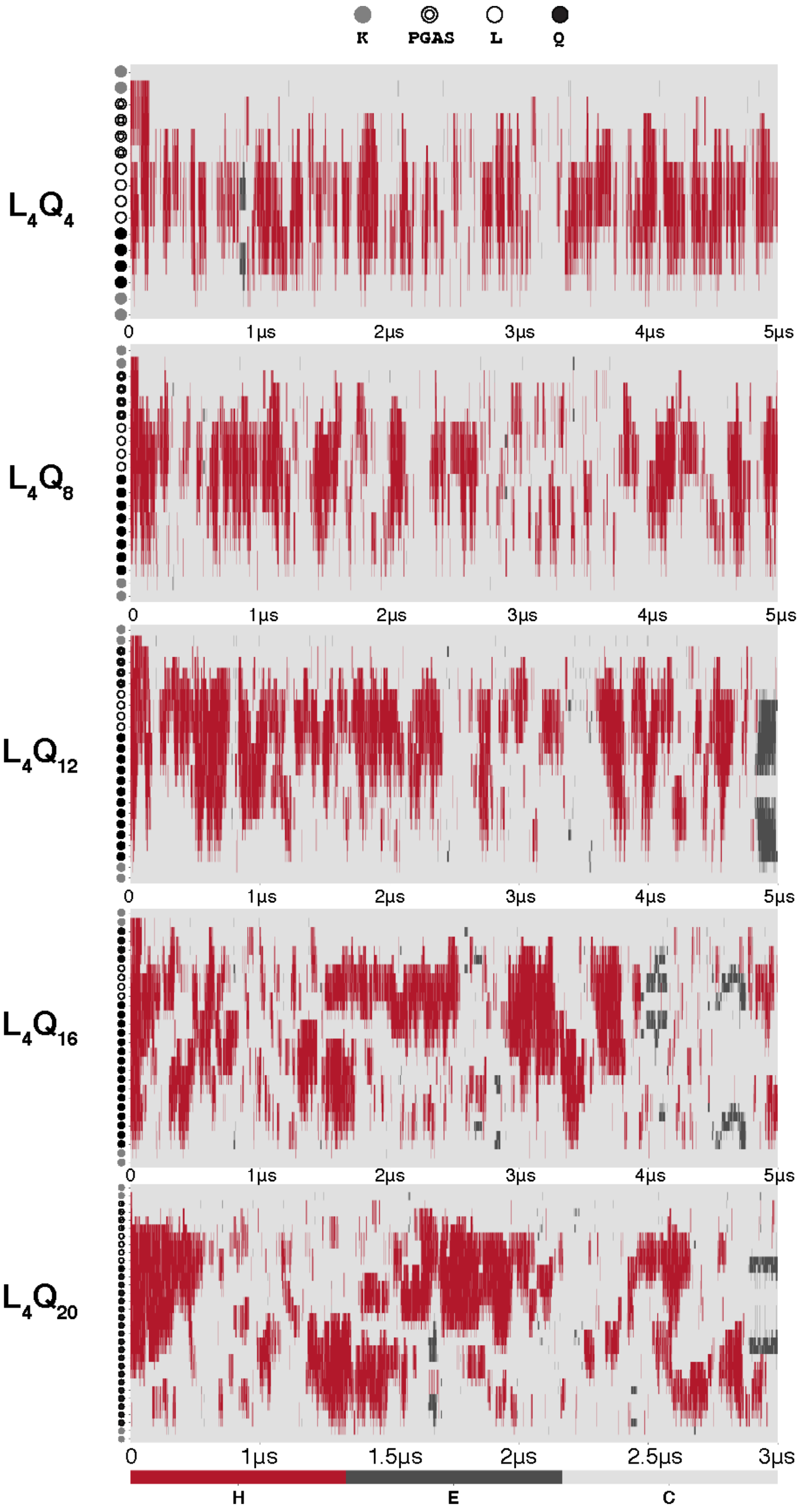

Supplementary Figure 7 | Time series of the secondary structure of peptides L_4Q_4 to L_4Q_{20} as obtained by using the algorithm DSSP [2] where H stands for helix, in red; E stands for extended, in dark gray; and C stands for coil, in light gray.

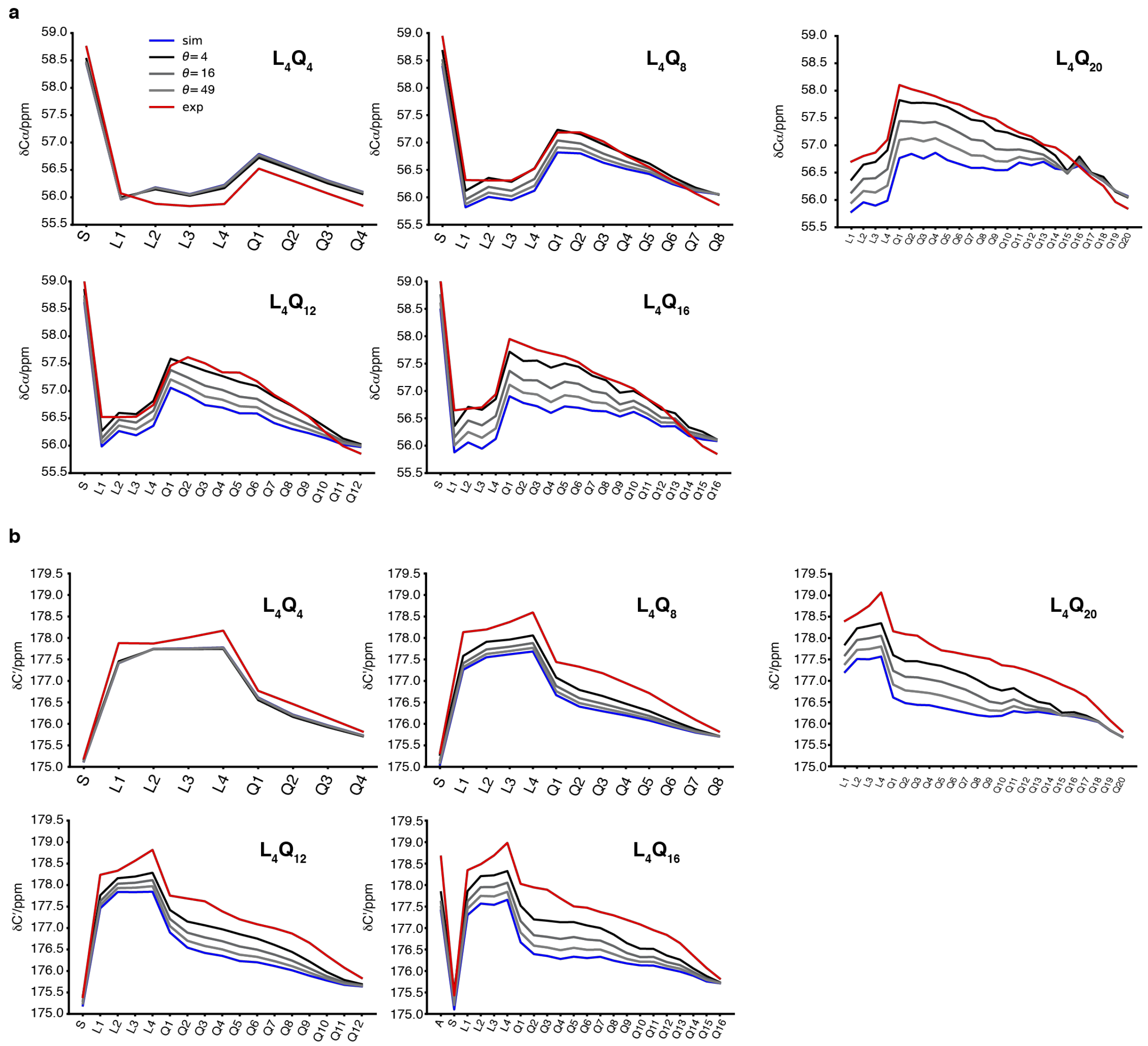

Supplementary Figure 8 | Experimental and back-calculated (a) Cα and (b) C' chemical shifts of peptides L_4Q_4 to L_4Q_{20} from the reweighted trajectories obtained with different values of the parameter θ, that determines the degree of reweighting applied in the ME algorithm.

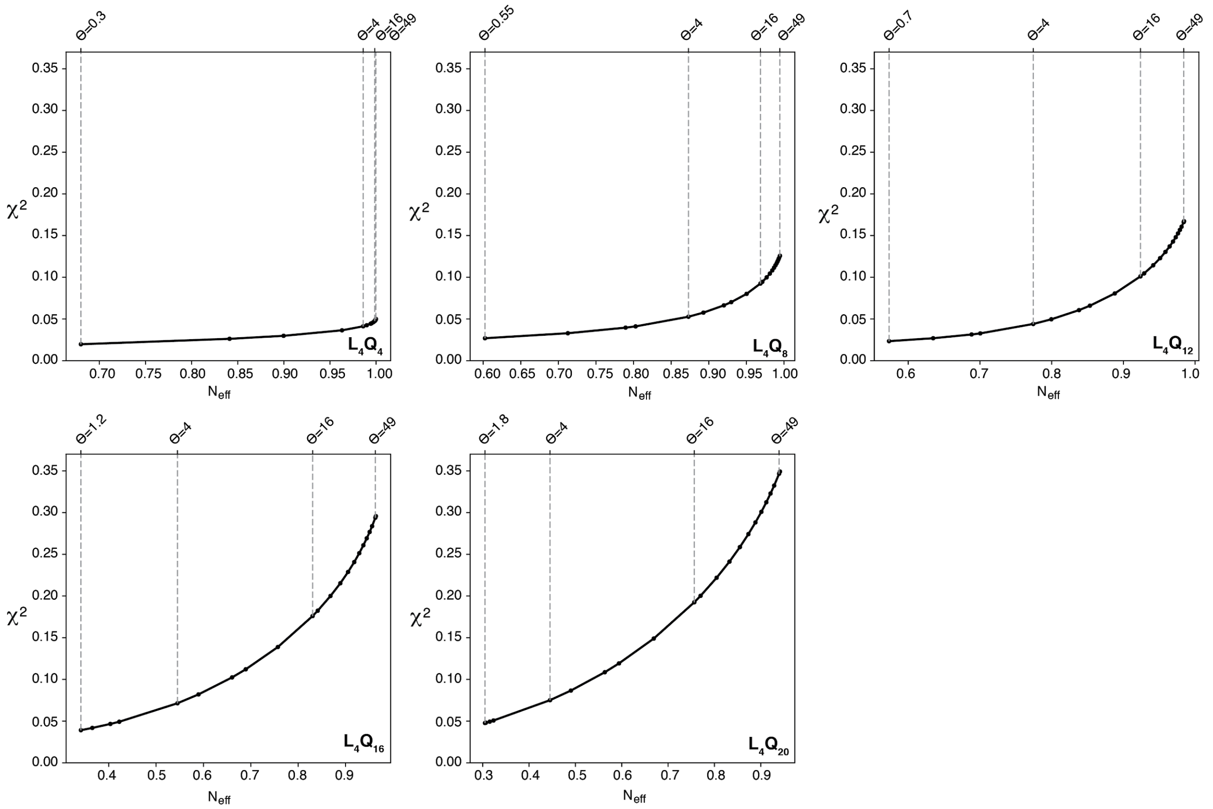

Supplementary Figure 9 | Effective fraction of frames after reweighting vs χ^2 for different values of θ. N_{eff} was calculated as $\exp(S_{rel})$ with S_{rel} being the relative entropy term in the BME reweighting approach. In short, N_{eff} quantifies the effective fraction of frames that are left after the trajectory has been reweighted to fit the data to an extent measured by χ^2.

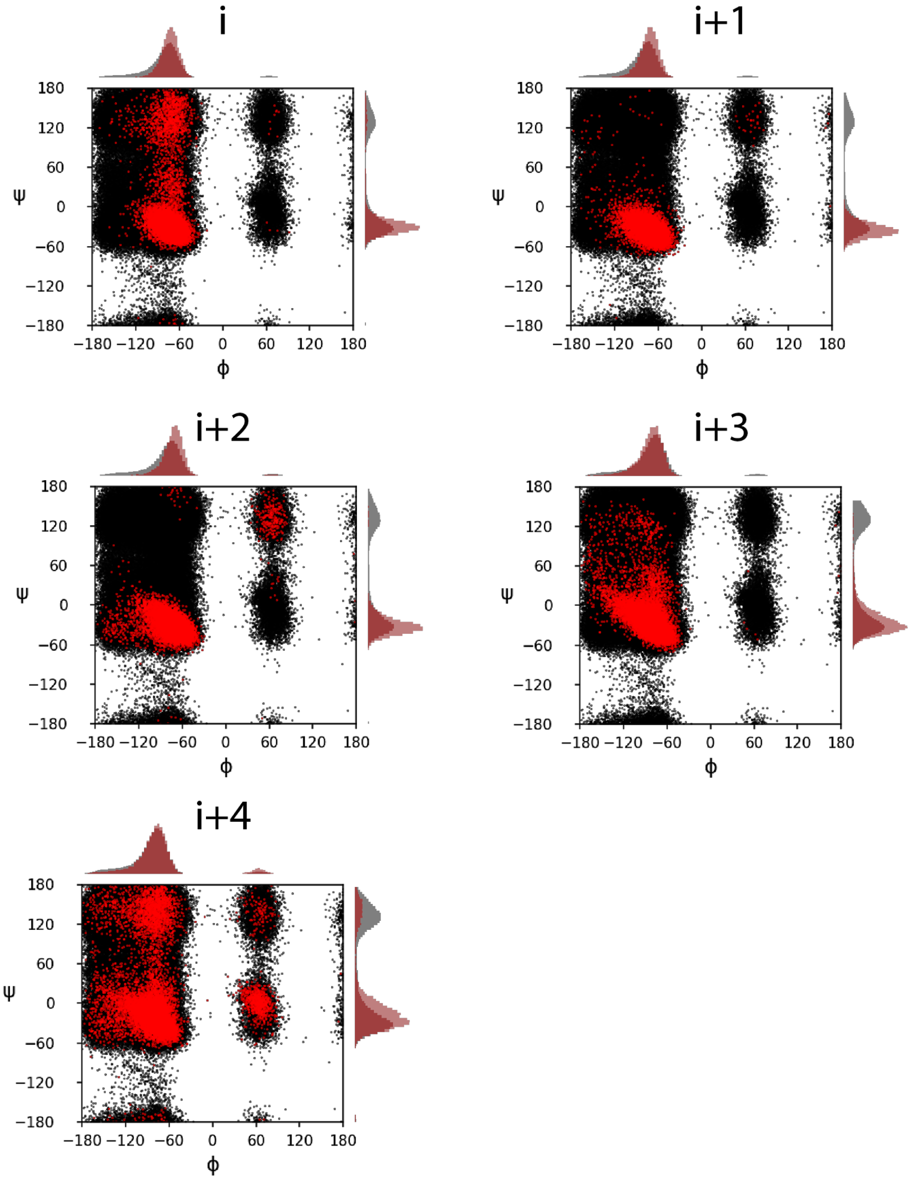

Supplementary Figure 10 | 2D histogram, in red, of backbone torsion angles φ and ψ for segments of five residues where the first and last residue are involved in a (i+4→i) side chain to main chain hydrogen bond. In black we show the result obtained when these residues are not involved in such interaction.

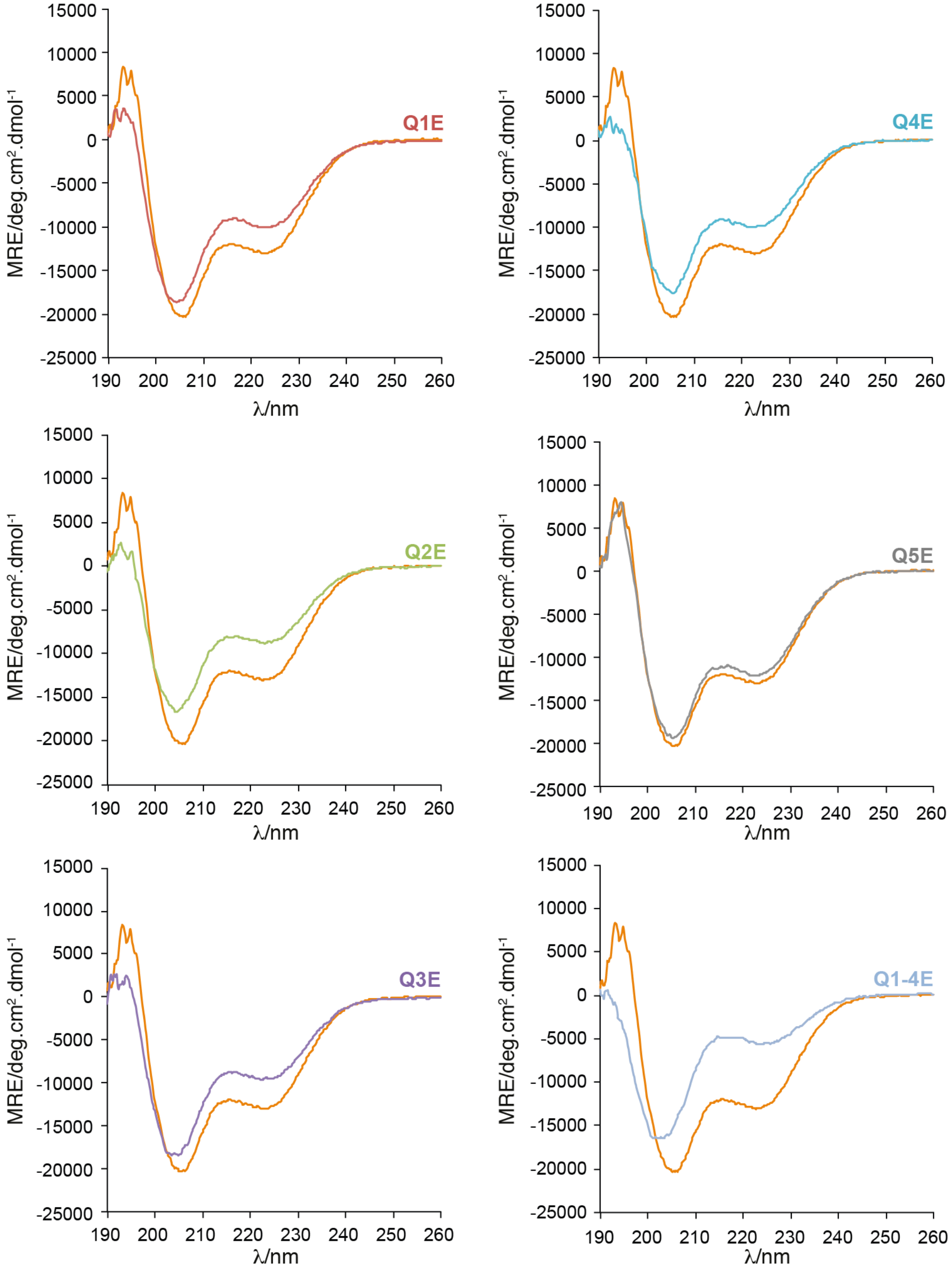

Supplementary Figure 11 | Comparison of the CD spectra of peptides Q1E to Q5E, as well as that of peptide Q1-4E, to that of peptide L_4Q_{16}, shown in orange in all plots.

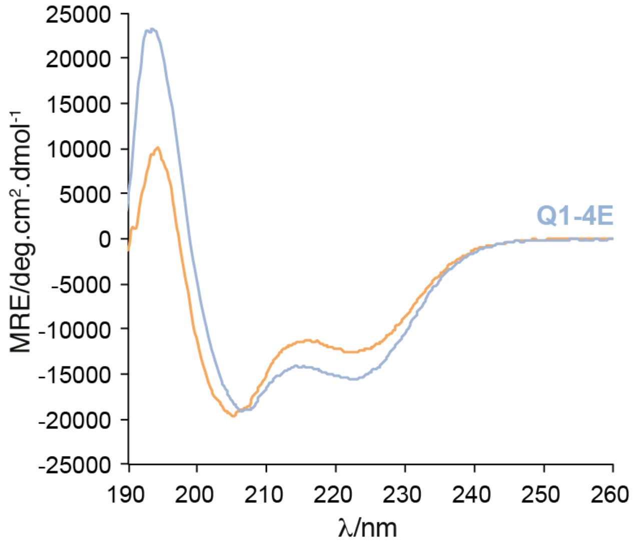

Supplementary Figure 12 | CD spectra of peptides L_4Q_{16} (in orange) and Q1-4E at pH 2

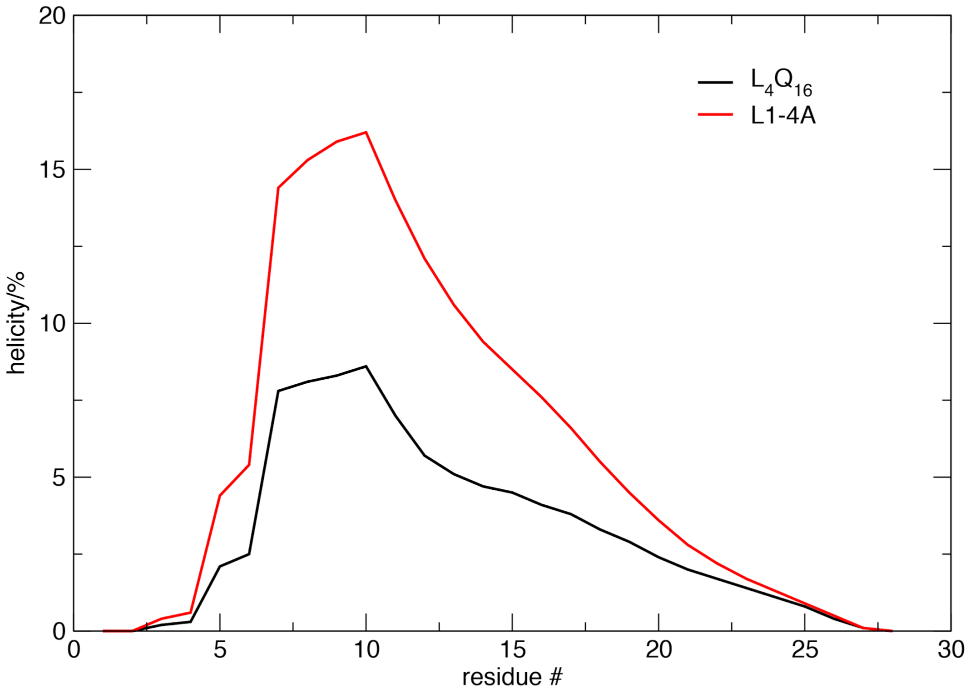

Supplementary Figure 13 | Prediction of the helicities of peptides L_4Q_{16} and L1-4A obtained by using Agadir[1].

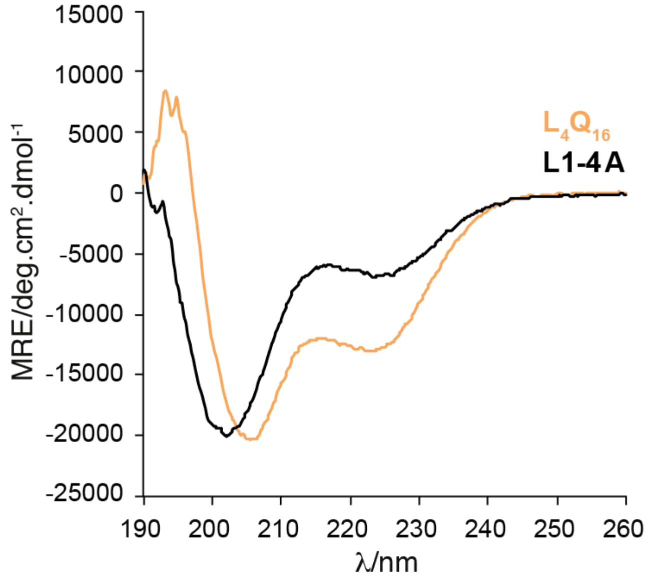

Supplementary Figure 14 | Comparison of the CD spectrum of peptide L1-4A, to that of peptide L_4Q_{16}.

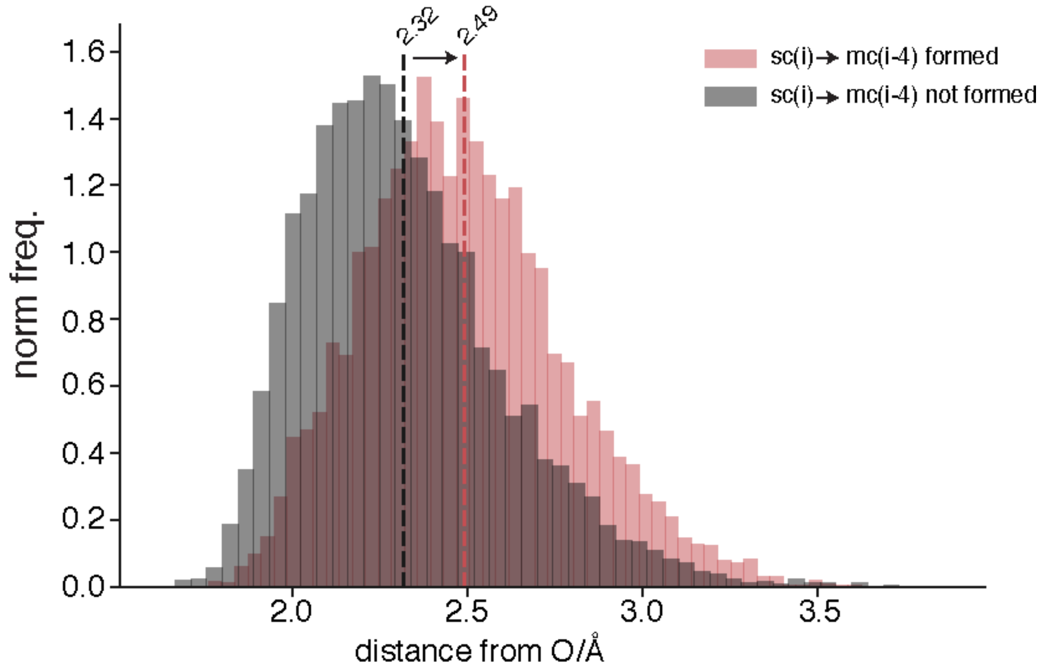

Supplementary Figure 15 | Distribution of the distance between the main chain NH of Q1 and the main chain CO of L1 in the absence and in the presence of the sc(Q1)→mc(L1) hydrogen bond.

L_4Q_4
ATGAGCGATAGCGAAGTTAATCAAGAAGCCAAACCGGAAGTTAAGCCGGAAGTGAAACCTG
AAACACATATTAACCTGAAAGTGAGTGATGGCAGCAGCGAAATCTTCTTCAAAATCAAAAAA
ACCACACCGCTGCGTCGTCTGATGGAAGCATTTGCAAAACGTCAGGGTAAAGAAATGGATA
GCCTGCGTTTTCTGTATGATGGTATTCGTATTCAGGCAGATCAGACACCGGAAGATCTGGAT
ATGGAAGATAACGATATTATCGAAGCACATCGTGAGCAGATTGGTGGTAAAAAACCGGGTG
CAAGCCTGCTGTTACTGCAGCAGCAACAGAAAAAATGATAA

L_4Q_8
ATGAGCGATAGCGAAGTTAATCAAGAAGCCAAACCGGAAGTTAAGCCGGAAGTGAAACCTG
AAACACATATTAACCTGAAAGTGAGTGATGGCAGCAGCGAAATCTTCTTCAAAATCAAAAAA
ACCACACCGCTGCGTCGTCTGATGGAAGCATTTGCAAAACGTCAGGGTAAAGAAATGGATA
GCCTGCGTTTTCTGTATGATGGTATTCGTATTCAGGCAGATCAGACACCGGAAGATCTGGAT
ATGGAAGATAACGATATTATCGAAGCACATCGTGAGCAGATTGGTGGTAAAAAACCGGGTG
CAAGCCTGCTGCTGCTTCAGCAGCAACAACAGCAACAGCAGAAAAAGTGATAA

L_4Q_{12}
ATGAGCGATAGCGAAGTTAATCAAGAAGCCAAACCGGAAGTTAAGCCGGAAGTGAAACCTG
AAACACATATTAACCTGAAAGTGAGTGATGGCAGCAGCGAAATCTTCTTCAAAATCAAAAAA
ACCACACCGCTGCGTCGTCTGATGGAAGCATTTGCAAAACGTCAGGGTAAAGAAATGGATA
GCCTGCGTTTTCTGTATGATGGTATTCGTATTCAGGCAGATCAGACACCGGAAGATCTGGAT
ATGGAAGATAACGATATTATCGAAGCACATCGTGAGCAGATTGGTGGTAAAAAACCGGGTG
CAAGCCTGCTGCTGCTTCAGCAGCAACAACAACAGCAACAGCAACAACAGCAGAAGAAGTG
ATAA

L_4Q_{16}
ATGAGCGATAGCGAAGTTAATCAAGAAGCCAAACCGGAAGTTAAGCCGGAAGTGAAACCTG
AAACACATATTAACCTGAAAGTGAGTGATGGCAGCAGCGAAATCTTCTTCAAAATCAAAAAA
ACCACACCGCTGCGTCGTCTGATGGAAGCATTTGCAAAACGTCAGGGTAAAGAAATGGATA
GCCTGCGTTTTCTGTATGATGGTATTCGTATTCAGGCAGATCAGACACCGGAAGATCTGGAT
ATGGAAGATAACGATATTATCGAAGCACATCGTGAGCAGATTGGTGGTAAAAAACCGGGTG
CAAGCCTGCTGCTGCTTCAGCAGCAACAACAGCAGCAACAGCAACAGCAACAGCAGCAACA
ACAGAAAAAGTAATAA

L_4Q_{20}
ATGAGCGATAGCGAAGTTAATCAAGAAGCCAAACCGGAAGTTAAGCCGGAAGTGAAACCTG
AAACACATATTAACCTGAAAGTGAGTGATGGCAGCAGCGAAATCTTCTTCAAAATCAAAAAA
ACCACACCGCTGCGTCGTCTGATGGAAGCATTTGCAAAACGTCAGGGTAAAGAAATGGATA
GCCTGCGTTTTCTGTATGATGGTATTCGTATTCAGGCAGATCAGACACCGGAAGATCTGGAT
ATGGAAGATAACGATATTATCGAAGCACATCGTGAGCAGATTGGTGGTAAAAAACCGGGTG
CAAGCCTGCTGCTGCTTCAGCAGCAACAACAGCAGCAACAGCAACAGCAGCAACAACAACA
ACAACAGCAGCAACAGAAGAAATGATAA

Supplementary Table 1 | Codon-optimized sequences used for the expression of peptides L_4Q_4 to L_4Q_{20}

Supplementary References

1. Muñoz, V. & Serrano, L. Elucidating the folding problem of helical peptides using empirical parameters. *Nat. Struct. Biol.* **1,** 399–409 (1994).
2. Kabsch, W. & Sander, C. Dictionary of protein secondary structure: pattern recognition of hydrogen-bonded and geometrical features. *Biopolymers* **22,** 2577–2637 (1983).
