# Supplementary material for: Side chain to main chain hydrogen bonds stabilize a polyglutamine helix in a transcription factor
Source: Nat Commun. 2019 May 2;10:2034. doi: 10.1038/s41467-019-09923-2 (PMC6497633; doi:10.1038/s41467-019-09923-2)
Supplement: Supplementary file 3 — Reporting Summary [file 41467_2019_9923_MOESM3_ESM.pdf]

nature research

Corresponding author(s): Ramon Crehuet, Kresten Lindorff-Larsen, Xavier Salvatella

Last updated by author(s): Apr 8, 2019

Reporting Summary

Nature Research wishes to improve the reproducibility of the work that we publish. This form provides structure for consistency and transparency in reporting. For further information on Nature Research policies, see Authors & Referees and the Editorial Policy Checklist.

Statistics

For all statistical analyses, confirm that the following items are present in the figure legend, table legend, main text, or Methods section.

n/a	Confirmed	
☒	☐	The exact sample size (*n*) for each experimental group/condition, given as a discrete number and unit of measurement
☒	☐	A statement on whether measurements were taken from distinct samples or whether the same sample was measured repeatedly
☒	☐	The statistical test(s) used AND whether they are one- or two-sided<br>*Only common tests should be described solely by name; describe more complex techniques in the Methods section.*
☒	☐	A description of all covariates tested
☒	☐	A description of any assumptions or corrections, such as tests of normality and adjustment for multiple comparisons
☒	☐	A full description of the statistical parameters including central tendency (e.g. means) or other basic estimates (e.g. regression coefficient) AND variation (e.g. standard deviation) or associated estimates of uncertainty (e.g. confidence intervals)
☒	☐	For null hypothesis testing, the test statistic (e.g. *F*, *t*, *r*) with confidence intervals, effect sizes, degrees of freedom and *P* value noted<br>*Give P values as exact values whenever suitable.*
☒	☐	For Bayesian analysis, information on the choice of priors and Markov chain Monte Carlo settings
☒	☐	For hierarchical and complex designs, identification of the appropriate level for tests and full reporting of outcomes
☒	☐	Estimates of effect sizes (e.g. Cohen's *d*, Pearson's *r*), indicating how they were calculated

Our web collection on statistics for biologists contains articles on many of the points above.

Software and code

Policy information about availability of computer code

Data collection	The CD data was collected with the software supplied by JASCO. The NMR data was collected withTOPSPIN (Bruker) and processed with nmrPipe and qMDD. The MD simulations were produced with AceMD. The QM/MM simulations were produced with AMBER interfaced to Terachem 1.9 (www.petachem.com). The Natural Bond Critical Point analysis was performed with NBO 6.0.
Data analysis	The CD data was analyzed with CONTIN in the website DICHROWEB (dichroweb.cryst.bbk.ac.uk/html/home.shtml). The NMR data was analyzed with CCPN Analysis and delta2D (www-vendruscolo.ch.cam.ac.uk). The MD trajectories were reweighted by using code available in GitHub (https://github.com/KULL-Centre/BME) by using PPM as chemical shift predictor (spin.ccic.ohio-state.edu/index.php/ppm). The secondary structure was analyzed by using DSSP (swift.cmbi.umcn.nl/gv/dssp).

For manuscripts utilizing custom algorithms or software that are central to the research but not yet described in published literature, software must be made available to editors/reviewers. We strongly encourage code deposition in a community repository (e.g. GitHub). See the Nature Research guidelines for submitting code & software for further information.

Data

Policy information about availability of data

All manuscripts must include a data availability statement. This statement should provide the following information, where applicable:

- Accession codes, unique identifiers, or web links for publicly available datasets
- A list of figures that have associated raw data
- A description of any restrictions on data availability

The chemical shifts obtained in this study and used for the generation of the conformational ensembles will be available in BMRB (www.bmrb.wisc.edu).

Field-specific reporting

Please select the one below that is the best fit for your research. If you are not sure, read the appropriate sections before making your selection.

☒ Life sciences ☐ Behavioural & social sciences ☐ Ecological, evolutionary & environmental sciences

For a reference copy of the document with all sections, see nature.com/documents/nr-reporting-summary-flat.pdf

Life sciences study design

All studies must disclose on these points even when the disclosure is negative.

Sample size	Does not apply to this work
Data exclusions	Does not apply to this work
Replication	All CD and NMR experiments were replicated.
Randomization	Does not apply to this study
Blinding	Does not apply to this study

Reporting for specific materials, systems and methods

We require information from authors about some types of materials, experimental systems and methods used in many studies. Here, indicate whether each material, system or method listed is relevant to your study. If you are not sure if a list item applies to your research, read the appropriate section before selecting a response.

Materials & experimental systems

n/a	Involved in the study	
☒	☐	Antibodies
☒	☐	Eukaryotic cell lines
☒	☐	Palaeontology
☒	☐	Animals and other organisms
☒	☐	Human research participants
☒	☐	Clinical data

Methods

n/a	Involved in the study	
☒	☐	ChIP-seq
☒	☐	Flow cytometry
☒	☐	MRI-based neuroimaging
